# Comparative Study on Electrical Conductivity of CeO_2_-Doped AlN Ceramics Sintered by Hot-Pressing and Spark Plasma Sintering

**DOI:** 10.3390/ma15072399

**Published:** 2022-03-24

**Authors:** Mickael Coëffe-Desvaux, Nicolas Pradeilles, Pascal Marchet, Marion Vandenhende, Mickael Joinet, Alexandre Maître

**Affiliations:** 1 IRCER UMR-CNRS 7315, 87068 Limoges, France; nicolas.pradeilles@unilim.fr (N.P.); pascal.marchet@unilim.fr (P.M.); marion.vandenhende@unilim.fr (M.V.); alexandre.maitre@unilim.fr (A.M.); 2 THALES AVS, 38430 Moirans, France; mickael.joinet@thalesgroup.com

**Keywords:** aluminum nitride (AlN), sintering aids, cerium oxide (CeO_2_), hot-pressing (HP), spark plasma sintering (SPS), electrical conductivity

## Abstract

Aluminum nitride (AlN) ceramics were prepared by both Hot-pressing (HP) and Spark-Plasma-Sintering (SPS) using cerium oxide as the sintering aid. The characterization of AlN raw powder denoted the presence of an amorphous layer that led to the formation of aluminum oxide. During the sintering process, CeO_2_ introduced as a sintering aid was reduced into Ce_2_O_3_. The latter reacted with aluminum oxide to form a transient liquid phase that promotes sintering by both HP and SPS. A reactional path leading to the formation of secondary phases, such as CeAlO_3_ and CeAl_11_O_18_, has been proposed according to the pseudo-binary Al_2_O_3_ – Ce_2_O_3_. Ceramics obtained from HP and SPS are presented as similar, except for the secondary-phase distribution. The influences of secondary phase composition and distribution on electrical conductivity were evaluated by leakage current measurements. The mechanism of DC conduction and the global conductivity of ceramics were discussed according to the sintering process and the number of secondary phases.

## 1. Introduction

Aluminum nitride ceramic (AlN) has been widely studied due to its interesting properties such as high thermal conductivity (up to 250 W m^−1^ K^−1^) [1,2], low coefficient of thermal expansion [3], low dielectric constant, and low loss tangent [4]. These properties make aluminum nitride a suitable material for electronic applications and a good candidate to replace traditional alumina or beryllium oxide as components for semiconductors equipment [5].

The full densification of the AlN has been reported as critical to control in order to improve targeted properties such as high thermal conductivity and high dielectric strength. The main problem arises during the densification process of AlN, due to the highly covalent nature of AlN bonds. To obtain highly dense ceramics, sintering must be carried out at high temperatures such as 1900 °C [6]. Consequently, pressure-assisted sintering processes have been studied to improve the kinetics of matter diffusion, and by these means, improve sintering, even at a lower temperature. Two technics gave promising results: Hot-Pressing (HP) and Spark-Plasma-Sintering (SPS) [7]. In a complementary way, using additives can be helpful to promote the densification of AlN at lower temperatures [8]. Thus, the literature provides plenty of examples of sintering aids. They could be divided into two groups: non-oxide additives such as CaF_2_ [6,9], and oxide additives. Rare-earth oxides are the most commonly used additive, such as Y_2_O_3_ [10,11], CeO_2_ [12], Sm_2_O_3_ [13,14], or La_2_O_3_ [15].

CeO_2_ has been studied as a sintering aid by Choi et al. [12]. AlN ceramics were obtained by pressure-less sintering with the addition of cerium oxide up to 1.50 wt.%. The authors observed improvements of properties such as thermal conductivity and hardness of the samples according to the increase in cerium oxide addition. This improvement is reported to be related to the increase in ceramics density.

The effects of CeO_2_ addition during hot-pressing sintering of aluminum nitride were explored by Li et al. [16]. In this article, an addition of 2 vol.% of CeO_2_ was compared with 2 vol.% of Y_2_O_3_ addition. CeO_2_ appeared to improve the density by about 1%, rather than yttrium oxide. Secondary phases observed by X-ray diffraction were not clearly identified but authors claimed that they were formed by a reaction between the rare-earth oxide and aluminum nitride. Once again, thermal properties and mechanical behavior improvements were only attributed to the improvement of density.

In an article based on the comparison between hot-pressing and pressure-less sintering of AlN, Jiang et al. [17] used a mixture of CeO_2_ and CeF_3_ as the sintering aid. They presented CeAlO_3_ as a secondary phase after sintering in addition to unreacted CeO_2_. A formation reaction of this cerium aluminate is presented (see Equation (1)).

(1) $$2{\mathrm{CeO}}_{2\left(s\right)}+{\mathrm{Al}}_{2}O_{3\left(s\right)}\;\to 2{\mathrm{CeAlO}}_{3\left(s\right)}+\frac{1}{2}O_{2\left(g\right)}↑$$

The thermal conductivity improvement was, this time, attributed to the decrease in oxygen impurities content through AlN ceramics. An extensive characterization of the dielectric responses of these ceramics pointed out the influence of both cerium-based additive and sintering processes.

Among research on sintering additives use, authors mainly focus on the improvement of final properties by increasing the densification of AlN ceramics [16]. It appears from the literature that both the sintering additive and sintering process played major roles in the improvement of aluminum nitride ceramics. However, limited information is available on the influence of the nature of secondary phases and their fraction on these properties’ modifications. In this study, new insights are presented in order to discriminate the influence of secondary phases from the influence of densification on the electrical properties of AlN ceramics. In recent decades, the SPS process has been presented as a promising method to obtain fully densified ceramics. Hence, it appeared useful in this work to compare the influence of this technic with a well-known technic such as HP. In this paper, both the sintering process and secondary phases’ fraction have been modulated in order to point out the role of the densification step on the final properties of AlN ceramics such as electrical conductivity.

## 2. Experimental Procedures

### 2.1. Raw Powders

Aluminum nitride powder (AlN, grade C, H.C. Starck, Munich, Germany) and cerium oxide powder (CeO_2_, ABCR, Karlsruhe, Germany) were used as starting materials. Chemical compositions of AlN and CeO_2_ powders were measured by Instrumental Gaz Analysis (IGA, Horiba, Kyoto, Japan) and Glow Discharge Mass Spectrometry (GDMS). The identification of crystalline phases within specimens was performed by means of an X-ray diffractometer (D8 Advance, Bruker, Bremen, Germany) in a Bragg–Brentano configuration using the Cu Kα1 wavelength (1.5406 Å, Johansson type monochromator, Ge (111), linear detector Lynxeye (Bruker, Bremen, Germany)). X-ray diffraction patterns were measured with a 20° to 80° 2θ angle, with a 0.01° step and a counting time of 0.5 s per step. For more visibility of secondary phases, most diffraction patterns are log-scale presented.

Particle size distributions of powders were determined by laser granulometry using a Mie scattering configuration after 30 s of in situ ultrasonic dispersion (Partica LA-950V2, Horiba, Kyoto, Japan).

Microstructures were characterized using (i) a scanning electron microscope with a BackScattered Electrons detector (BSD) (18 nm carbon coating, SEM-FEG, LEO 1530 VP, ZEISS, Oberkochen, Germany), (ii) a second scanning electron microscope coupled with a focused ion beam (FEG-SEM-FIB with BSD, Zeiss Crossbeam 550, ZEISS, Oberkochen, Germany), and (iii) a transmission electron microscope (JEOL 2100F, JEOL, Tokyo, Japan).

Sintered samples are composed of AlN with 1 wt.% and 3 wt.% of CeO_2_, respectively. The powder mixtures were performed in ethanol using a planetary mill (Fritsch, Idar-Oberstein, Germany) with a corundum jar and balls (jar filled to its 2/3 point with a powder–ball–ethanol volume ratio of 1:1:1). The milling process is composed of 1 min of milling at 250 rpm followed by a 3 min pause to avoid heating. This sequence is repeated 15 times. This milling step is mandatory to obtain a homogeneous mix of the two powders, improving the sintering ability. The obtained slurry was dried at 70 °C for 12 h.

### 2.2. Sintering and Characterizations

Sintering was performed in 30-mm-diameter graphite die with Papyex^®^ (MERSEN, Paris, France) graphite sheets to protect the die. Hot-pressing was performed using a Goliath Hot-Press (LPA, Paris, France) at 1850 °C with a uniaxial load of 30 MPa under nitrogen flow (<2 ppm of O_2_). The ramps were set at 10 °C/min, and a dwell of 90 min was chosen. Spark plasma sintering, performed using a Dr Sinter 825 SPS (Fuji Electronics Co. Ltd, Tokyo, Japan), was conducted under a nitrogen atmosphere at 30 MPa load, up to 1650 °C with a 100 °C min^−1^ heating ramp, a dwell of 15 min, and a cooling rate of 50 °C min^−1^. The selected sintering parameters (on HP and SPS) allowed us to obtain equivalent samples from the density and grain size perspectives.

The density of samples was determined according to the Archimedes principle in absolute ethanol at a controlled temperature.

Thermal analyses were carried out using a thermogravimetric device (SETSYS TGA, Setaram Instrumentations, Caluire-et-Cuire, France) coupled to an original quenching configuration. This device allows one to quickly extract the sample from the hot zone. The cooling rate typically achievable is about 1000 K min^−1^. These analyses were performed under a flow of nitrogen (H_2_O <3 ppm; C_n_H_m_ <0.5ppm and O_2_ <2 ppm) up to different temperatures (1400 °C and 1500 °C) with a heating rate of 10 °C min^−1^. Thanks to this device, a previous study elucidated the formation mechanism of yttrium aluminates during the sintering of aluminum nitride with yttrium oxide as a sintering aid [18].

Electrical behavior was determined by leakage current measurement in a TF analyzer (2000-PSHU, AixACCT, Aachen, Germany) by measuring the current going through the sample bulk under voltages varying between 0 kV and 2 kV. The samples (1 mm thick and 5 mm square) were metalized by a silver coating on the upper and lower faces and placed in silicon dielectric oil. The temperature variation could be controlled from 25 °C to 250 °C by 25 °C steps.

## 3. Results

### 3.1. Raw Powders Characterizations

The morphological features and the chemical composition of raw powders were characterized to evidence the most critical parameters that can influence their reactivity during heating.

**Figure 1:**
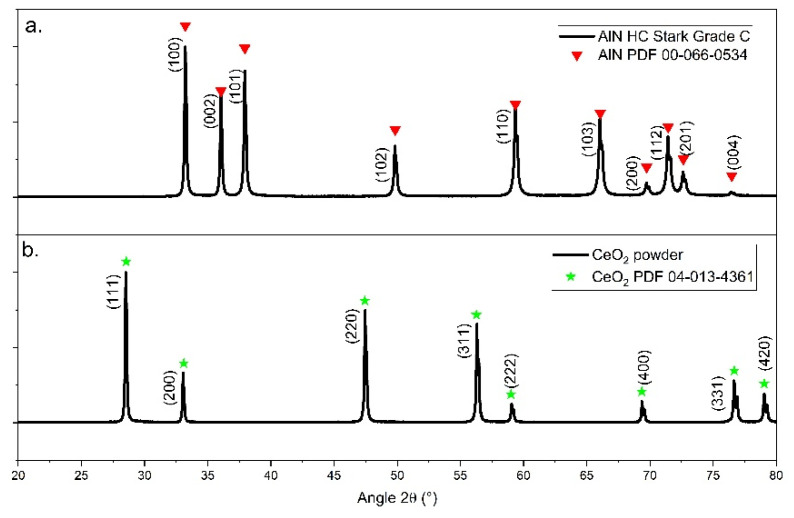
X-ray diffraction patterns of (**a**) aluminum nitride raw powder; (**b**) cerium oxide raw powder.

X-ray diffraction patterns (XRD) of the raw powders in Figure 1 permitted us to assume that hexagonal AlN, identified from PDF 00-066-0534, and cubic CeO_2_, identified from PDF 04-013-4361, are, respectively, the only crystallized phase in each raw powder.

Chemical analyses were conducted in order to determine the purity of the compounds. Glow discharge mass spectrometry (GDMS) and instrumental gas analysis (IGA) were performed in order to identify most of the impurities within the powders. As presented in Table 1, impurities within cerium oxide powder have been detected, such as phosphorus, aluminum, or sodium, in reasonable quantities for its use as a sintering aid. Aluminum nitride powder presented few impurities, besides the presence of a substantial amount of oxygen and some hydrogen detected by IGA. As no additional crystallized phase is present (by XRD analysis), oxygen and hydrogen can be related to amorphous surface oxidation and/or hydroxylation such as amorphous alumina, Al_2_O_3_, or boehmite-like phases, AlO(OH).

**Figure 2:**
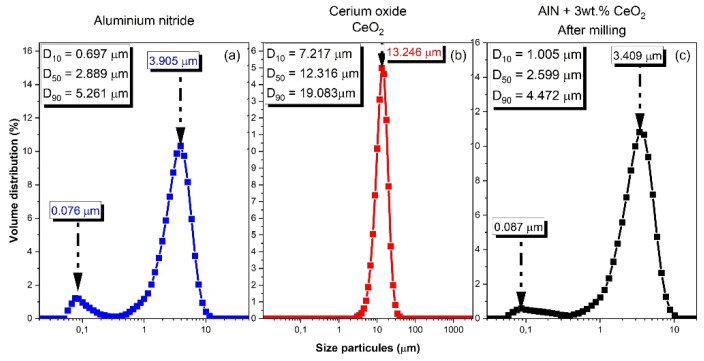
Particle size distribution of (**a**) aluminum nitride raw powder; (**b**) yttrium oxide raw powder; (**c**) AlN + 3 wt.% CeO_2_ after milling.

**Figure 3:**
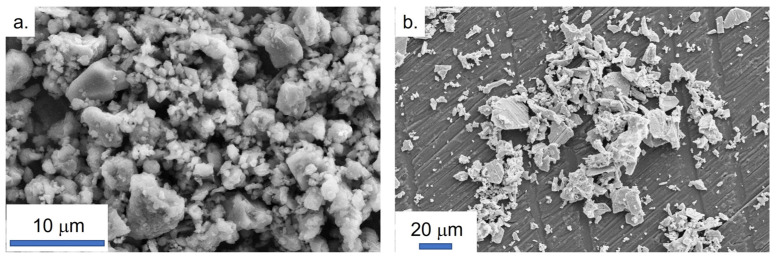
SEM observations of the raw powders. (**a**) Aluminum nitride; (**b**) cerium oxide.

AlN powder shows a bimodal particle size distribution with the first population centered on 80 nm and the second one exhibiting an average value of 3.905 μm (see Figure 2). These data are lightly different from those given by the powder supplier. The difference has been explained by the agglomeration of small particles around bigger ones as observed by SEM in Figure 3a. The so-formed aggregates could be spears during the milling process. As observed in Figure 2b and confirmed by the analyses presented in Figure 3b, ceria powder presents larger particles than AlN powder. The average particle size is centered on 13.246 µm.

The milled samples showed a final granulometry composed of two populations, as represented in Figure 2c. The first one is centered on 3.409 µm and the second one is centered on 0.087 µm. The milling process allows one to decrease the grain size of cerium oxide powder and permits one to obtain a homogenized powder composed of AlN and a sintering additive.

### 3.2. Specific Focus on Aluminium Nitride Powder

**Figure 4:**
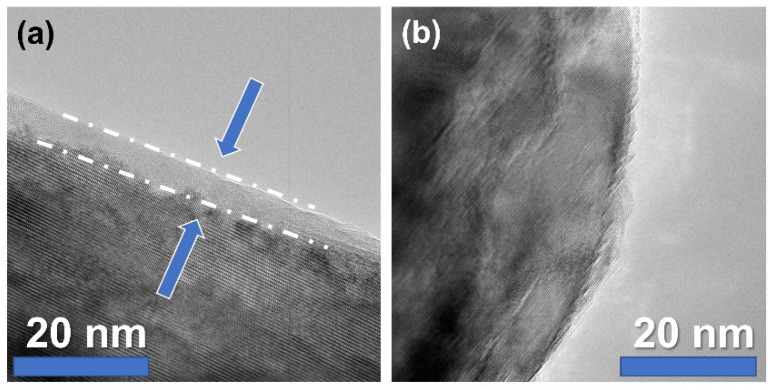
TEM observations of an AlN grain. The arrow points to a nanosized-thick amorphous layer. (**a**) AlN particle with amorphous layer, (**b**) AlN particle without amorphous layer.

To delve deeper into the AlN powder analyses, examinations were conducted by transmission electronic microscopy (TEM) to obtain further information on the oxidation detected by IGA. As presented in Figure 4a, a 2–5-nm-thick amorphous layer can be observed by TEM and is assumed to be uncrystallized aluminum oxide.

**Figure 5:**
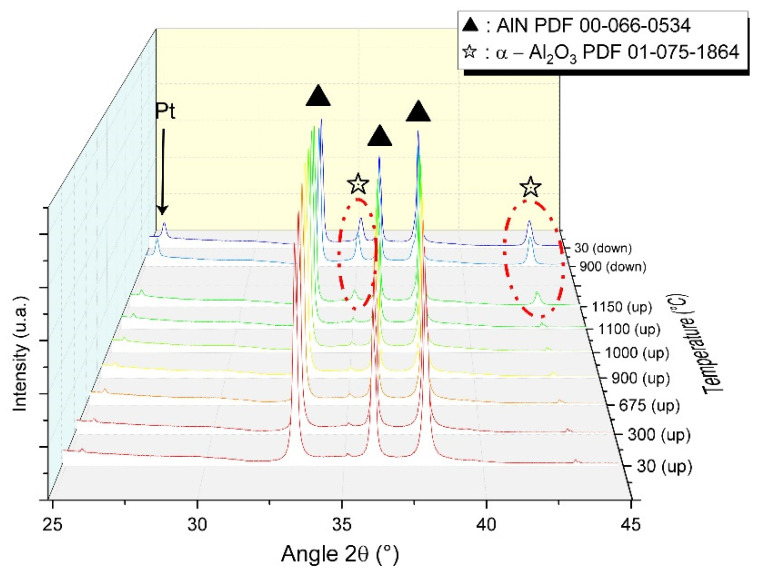
X-ray diffraction patterns of aluminum nitride (AlN) powder during in situ thermal treatment under N_2_ atmosphere.

During sintering, this amorphous phase is supposed to play a key role in the formation of secondary phases. In order to determine how it affects densification, the AlN raw powder was thermally treated under nitrogen to identify possible phase transformation. An in situ analysis was performed by X-ray diffraction during thermal treatment up to 1150 °C. The diffraction pattern evolution is presented in Figure 5.

The powder is placed on a platinum foil also used as an internal reference for thermal expansion of the system. Small diffraction peaks of alumina are detected during the heating step from room temperature to 1100 °C. The latter is assumed to come from the sample holder. However, from 1150 °C, corundum peaks were strongly intensified. During cooling, these peaks remained strong.

**Figure 6:**
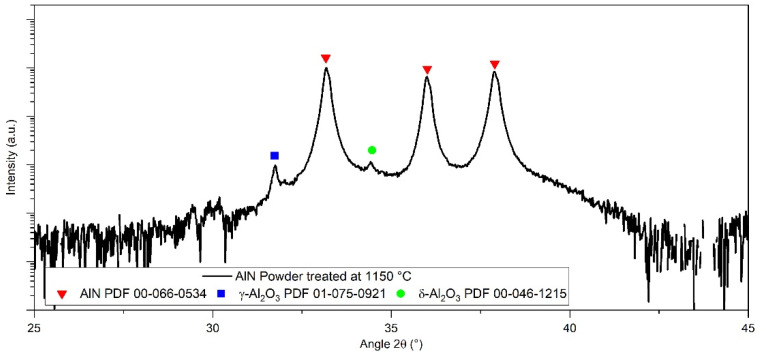
Selected area of AlN treated at 1150 °C diffraction pattern.

A post-treatment analysis was conducted outside of the XRD device in order to confirm the crystallization of alpha-aluminum oxide during the heating of AlN powder. The XRD pattern of the treated powder is presented in Figure 6.

It appeared that the powder is mainly composed of aluminum nitride and some traces of γ and δ aluminum oxide. This observation will be developed in the discussion section.

### 3.3. Densification and Microstructure of CeO_2_ Doped Aluminium Nitride Ceramics

In order to compare the electrical properties of aluminum nitride ceramics doped with cerium oxide, it is necessary to obtain samples with both an equivalent density and an equivalent microstructure. Samples sintered by HP were selected as the reference as a result of their high densification rate, low open porosity fraction, and average grain size.

**Figure 7:**
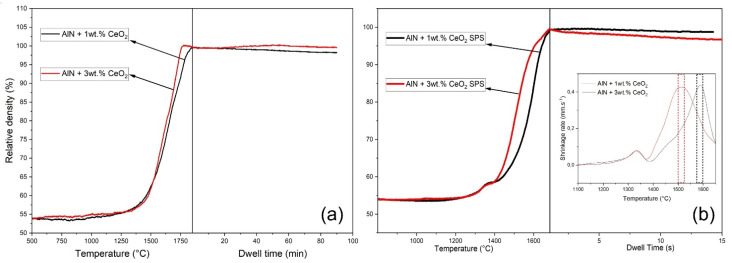
Sintering curves of samples with 1 wt.% and 3 wt.% obtained by (**a**) hot-pressing and (**b**) spark plasma sintering.

Sintering curves obtained by both hot-pressing and spark plasma sintering are reported in Figure 7.

The temperatures for sintering are reported in Table 2. As observed, sintering by SPS occurred at a different temperature than HP. The difference in the observed temperatures is related to the process itself.

As observed in Figure 7b, during SPS treatment, the first thermal event occurred at the early stage of sintering, above 1360 °C. This event has not been observed during HP treatment. SPS, because of its high heating rate, can exacerbate phenomena such as liquid phase formation. This first inflexion observed between 1300 °C and 1350 °C could be a sign of the liquid phase forming out of the thermodynamic equilibrium. During hot-pressing, the sample is always considered close to the thermodynamic equilibrium, as a result of the slow heating. The influence of cerium oxide and sintering technic will be presented in the Discussion section.

Table 3 presents the density of samples obtained with 1 wt.% and 3 wt.% of cerium oxide and sintered by HP and SPS, respectively. Samples with an identical fraction of the sintering additive present the same density after sintering. For all samples presented, the open porosity fraction was measured below 0.5%. When the fraction of the sintering aid increases, the absolute density of the ceramic increases as well. According to the secondary phases identified and presented below, the relative density of each sample has been calculated as higher than 99%. Hence, the densification of the so-obtained ceramics is not dependent on the amount of the sintering aid. A well-optimized sintering cycle allows one to densify doped AlN up to the theoretical density with a low amount of the sintering aid.

**Figure 8:**
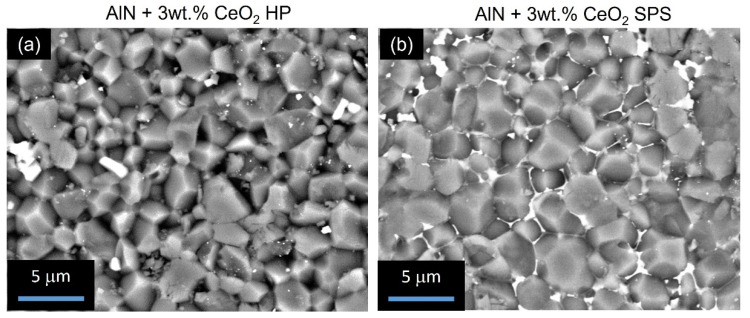
BSD-SEM observations of fractured surface of samples composed of 3 wt.% of cerium oxide (**a**) sintered by HP, (**b**) sintered by SPS.

Microstructures of aluminum nitride doped with 3 wt.% of cerium oxide, sintered by HP and SPS, have been observed by SEM. Figure 8 presents fractured surfaces of these ceramics, obtained by the backscattered electron mode. Microstructures generated by HP and SPS appeared to be substantially equivalent according to grain size observations. Thus, it confirmed that the SPS cycle permits one to obtain a microstructure equivalent to HP-sintered ceramics. However, the spatial distribution of the secondary phases appeared to be quite different from these two samples. In the hot-pressed sample, the secondary phases are located at the triple point of the grain boundaries. These phases exhibited a grain-like shape when they agglomerated. The sample obtained by SPS presents secondary phases located all over grain boundaries. The secondary phases form a continuous network around AlN grains. However, some secondary phases appear agglomerated at the triple point as observed for the HP sample.

**Figure 9:**
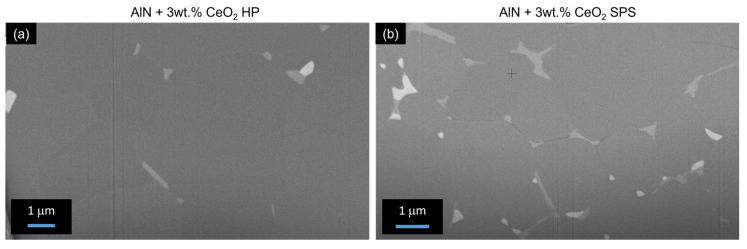
Microstructure of AlN + 3wt.% CeO_2_ samples polished in situ by SEM-FIB. (**a**) sample sintered by HP, (**b**) sample sintered by SPS.

Complementary SEM-FIB observations were made in order to confirm the presence of a continuous network of secondary phases. The surfaces of the samples doped with 3 wt.% and sintered by HP and SPS, respectively, have been polished by an in situ focused gallium ion beam. SEM observations are reported in Figure 9. First, it can be noted that no porosity was observed, confirming the results obtained by Archimedes’ measurements. As observed in Figure 8, the secondary phases of the HP sample are confirmed at the grain’s triple points, mainly agglomerated. Because secondary-phase grains are not homogeneously dispersed around AlN grains, it appeared difficult to obtain good observations of secondary phases by SEM-FIB for this sample. On the other hand, secondary phases are easily observed for the SPS sample. It appeared that these phases did not form a continuous percolated network around AlN grains but are well-dispersed around them.

**Figure 10:**
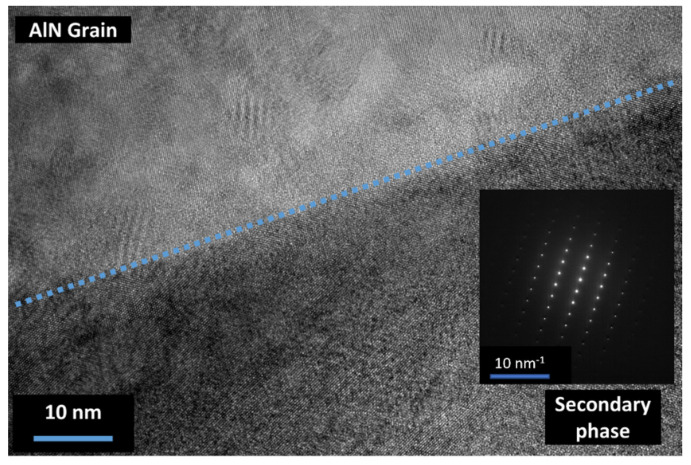
Observation by TEM of a grain boundary of AlN + 3 wt.% CeO_2_ sintered by SPS.

TEM observations were conducted in order to determine the nature of secondary phases. As observed in Figure 10, the secondary phases located at the grain boundary appeared fully crystallized. However, it was not possible to make a full indexation of the diffraction pattern. Extensive characterization is needed on this topic. No evidence of an amorphous phase at grain boundaries has been underlined from TEM examinations.

### 3.4. Secondary Phase Formation

**Figure 11:**
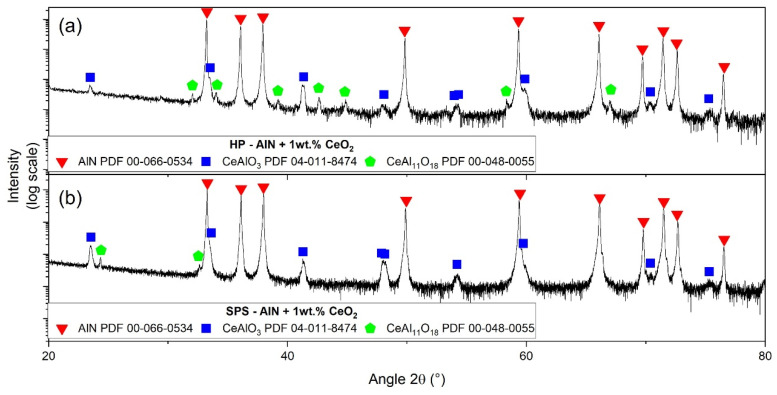
X-ray diffraction patterns of AlN + 1 wt.% of cerium oxide obtained by (**a**) HP and (**b**) SPS.

Secondary phases that formed after sintering were identified by X-ray diffraction for samples obtained by HP and SPS. The characterizations performed for samples composed of 1 wt.% of ceria are presented in Figure 11. After sintering, the main secondary phase is cerium aluminate CeAlO_3_. A second crystalized phase was detected in minute traces formed by CeAl_11_O_18_, the second defined compound in the Al_2_O_3_–Ce_2_O_3_ pseudo-binary system. Regardless of how the samples were sintered, the secondary phases exhibited by XRD analysis were the same.

**Figure 12:**
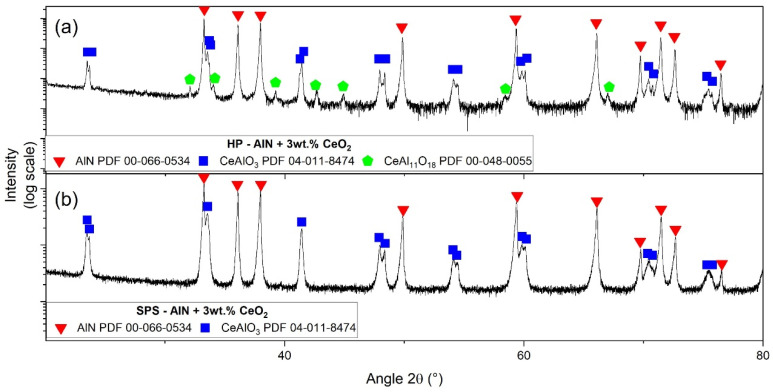
X-ray diffraction patterns of AlN + 3 wt.% of cerium oxide obtained by (**a**) HP and (**b**) SPS.

Figure 12 presents XRD patterns of AlN + 3 wt.% CeO_2_ samples sintered by hot-pressing and spark plasma sintering, respectively. Similar to samples obtained with 1 wt.% of CeO_2_, CeAlO_3_ is the main secondary phase detected. However, a difference appeared between the HP sample and the SPS sample. Traces of CeAl_11_O_18_ disappeared from the SPS sample.

**Figure 13:**
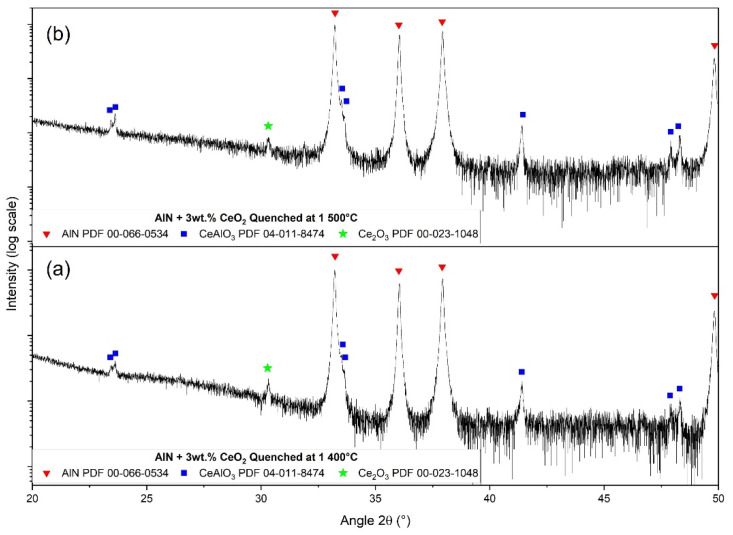
X-ray diffraction patterns of AlN + 3 wt.% CeO_2_ samples quenched under nitrogen at (**a**) 1400 °C, (**b**) 1500 °C.

In order to identify the reactions leading to the transformation of CeO_2_ to cerium aluminates, quenching tests were conducted. Samples of AlN + 3 wt.% CeO_2_ were heated to the target temperature under a N_2_ protective flow and quenched by extracting the sample from the hot zone of the device. X-ray diffraction analysis was performed on the so-obtained samples in order to identify the frozen intermediate phases generated. Results are presented in Figure 13.

The two quenching temperatures produced a rather similar result. Crystalized CeAlO_3_ was identified, such as cerium sesquioxide Ce_2_O_3_. No remaining traces of CeO_2_ were detected. The formation mechanisms of secondary phases are presented in the Discussion section.

### 3.5. Electrical Properties of Sintered Ceramics

**Figure 14:**
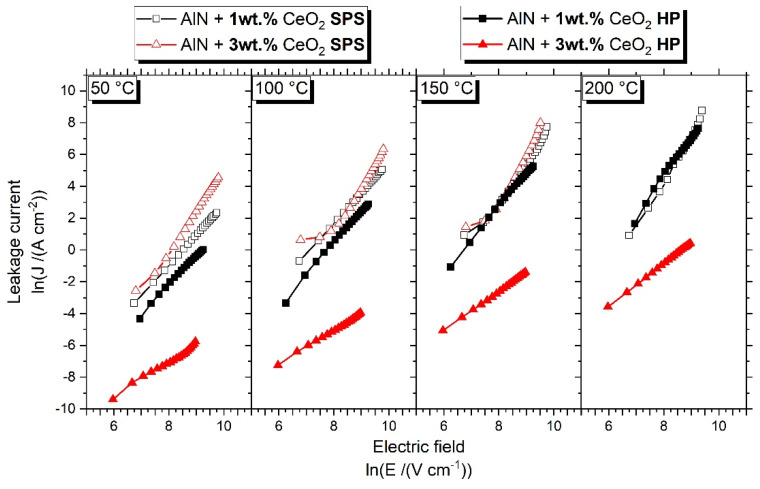
Leakage current measurement of HP and SPS samples.

The electrical behavior of sintered ceramics was determined as a function of the temperature by measuring the leakage current passing through the volume of the sample under an applied electrical field. The conduction mechanism of aluminum nitride has been attributed to Space Charge Limited Current (SCLC) by numerous authors [19,20]. Leakage current measurement (LM) allowed for determining the mode of conduction according to the SCLC mechanism as a function of the applied electrical field and temperature. Figure 14 presents, in a log–log representation, leakage current measurement obtained for the samples sintered by HP and SPS, from 50 °C to 200 °C.

As observed in Figure 14, the mechanism leading to current generation within the ceramic volume is rather similar between HP and SPS samples with 1 wt.% cerium oxide addition. The sample doped with 3 wt.% of CeO_2_ and sintered by SPS presented similar behavior to the 1 wt.% samples. However, it was not possible to obtain data at 200 °C. The conduction through this sample reached the measurement limit when the temperature increased up to 150 °C. The sample sintered by HP with 3 wt.% CeO_2_ is different from the others. It shows, at the presented temperatures, lower values of leakage current, meaning the sample is more resistive than the other three.

As described by the SCLC model, a log–log graphic allows one to identify the electrical behavior of the sample according to the measured slopes in this representation. Table 4 summarizes the slopes extracted from Figure 14.

Slopes appeared to be rather similar for the samples doped with 1 wt.% CeO_2_. Two slopes were identified at 150 °C and 200 °C for the SPS sample. The first one of each temperature, measured at low field and denoted as (L), is close to the slope measured for the HP sample. However, the second one, denoted as (H) and measured at a high electric field, is higher than the HP sample. This observation denotes a change in the conduction mechanism according to the sintering technic, which will be developed in the discussion section.

The results of leakage current measurement for AlN + 3 wt.% samples sintered by HP and SPS are rather different. The HP-sintered sample presents slopes around 1 for the three measured temperatures. The ceramic obtained by SPS presents higher slopes, with values of approximately 3. The difference in leakage current measured between HP and SPS samples shows an important variation of conductivity between these two samples.

**Figure 15:**
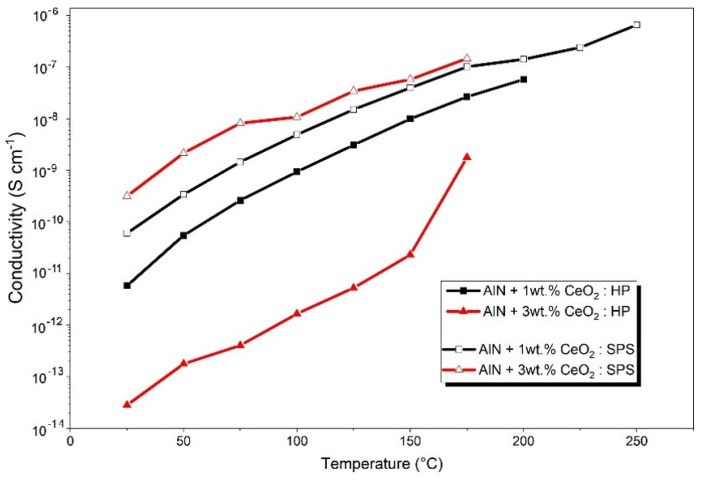
Thermal evolution of the global conductivity of AlN + 1wt.% CeO_2_ and AlN + 3wt.% CeO_2_. Comparison between HP and SPS samples.

Leakage current measurement permits one to determine the global conductivity of the samples. Thermal evolutions of electrical conductivity of cerium-oxide-doped aluminum nitride sintered by HP and SPS are presented in Figure 15. The conductivity of all sintered ceramics increases several orders of magnitude with the temperature, regardless of the amount of sintering additive or the sintering process. According to Figure 15, thermal evolutions of the electrical conductivity of samples doped with 1 wt.% CeO_2_ are rather similar. The difference remains in the value of conductivity, which is one order of magnitude higher for the sample sintered by SPS. As pointed out by Figure 15, samples composed of 3 wt.% CeO_2_ addition presented a large gap in conductivity values between HP and SPS. The largest measured difference is about 10^4^ S cm^−1^. Regarding the AlN + 1 wt.% CeO_2_ sample sintered by HP, the ceramic obtained by the same sintering process with 3 wt.% of CeO_2_ appeared to be much more resistive. The difference between compositions with 1 wt.% and 3 wt.% of the additive sintered by SPS is not as significant. 

## 4. Discussions

### 4.1. Sintering of CeO_2_ Doped Aluminium Nitride Ceramics

It is mainly accepted that the sintering of aluminum nitride with sintering aids generates a transient liquid phase that promotes densification by increasing the mass transfer via diffusion [21]. However, limited information is given on the formation of this liquid phase. Extensive characterization of AlN raw powder, such as the TEM presented in Figure 4, permitted us to point out an amorphous phase remaining on the AlN grains’ surface. This phase is assumed to be an amorphous aluminum oxyhydroxide that will crystalize during thermal treatment. This kind of amorphous phase around non-oxide ceramics has already been observed for AlN [22] and some other non-oxide ceramics such as B_4_C [23] or ZrC [24]. According to Figure 5 and Figure 6, it can be assumed that this phase will follow several phase transformations leading to the formation of transition alumina such as γ-Al_2_O_3_, δ-Al_2_O_3_, and, finally, α-Al_2_O_3_. These phase transformations have already been observed during the dehydration process of boehmite phases [25].

**Figure 16:**
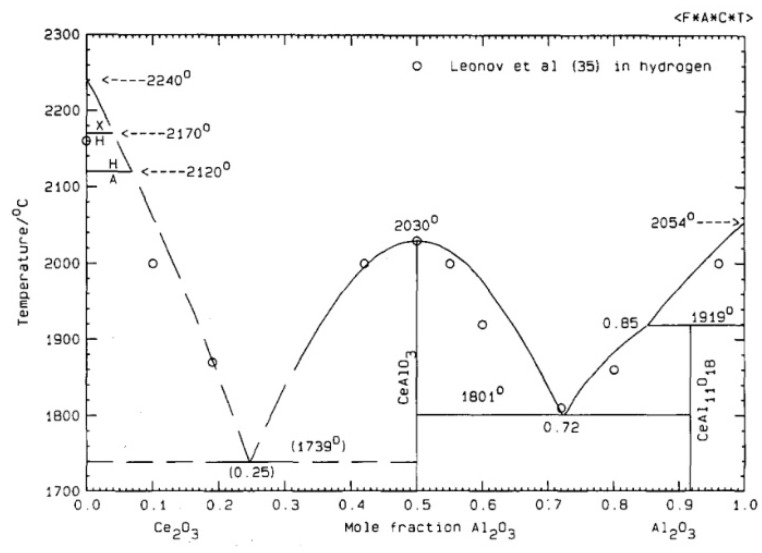
Pseudo-binary phase diagram of aluminum oxide and cerium oxide [26]. Reprinted from Journal of Alloys and Compounds, Vol 179, P. Wu and A.D. Pelton, “Coupled thermodynamic-phase diagram assessment of the rare earth oxide-aluminium oxide binary systems”, pages 264, Copyright (1992), with permission from Elsevier.

Regarding the pseudo-binary Al_2_O_3_–Ce_2_O_3_, in Figure 16, the formation of liquid is allowed at 1750 °C and 1800 °C, the two eutectic points. Hence, it confirms the possible formation of a liquid during sintering involving the sintering aid, CeO_2_, and alumina formed during the thermal treatment of AlN powder (from the amorphous layer). The liquid phase generated during sintering promotes densification and also enhances the formation of the secondary phase by homogenizing the global grain boundaries’ composition. The addition of 3 wt.% of cerium oxide during HP sintering allowed it to reach full densification at a lower temperature than with 1 wt.%. This improvement can be attributed to the volume fraction of secondary phases generated during thermal treatment. A higher amount of the transient liquid phase led to an increase in the homogeneity of grain boundaries, and as a result, enhanced the kinetic of densification. During HP sintering, the formation of this transient liquid phase could not be clearly identified according to shrinkage curves. However, because SPS allows fast heating, and hence generated a high thermal gradient within the sample, it is possible to exacerbate some phenomena by placing the sample in a thermodynamic non-equilibrium state [23]. In Figure 7b, the first step of densification is observed around 1360 °C. The beginning of shrinkage can be attributed to the formation of this liquid phase, in the same way as it was observed during the sintering of aluminum nitride with yttrium oxide as a sintering aid [18,22]. At this temperature, the formation of a liquid phase can be explained by either (i) the presence of incomplete crystallized alumina or polymorphous phases (see Figure 6), reducing the formation energy of the liquid, (ii) the SPS technic that generates a high thermal gradient due to fast heating (100 °C min^−1^). Hence, during the SPS treatment of some ceramics and, more precisely, semi-conductors, the temperature measured at the matrix surface could appear lower than the inner temperature of the sample [23].

According to the pseudo-binary Al_2_O_3_–Ce_2_O_3_, only two defined compounds could be formed: CeAlO_3_ and CeAl_11_O_18_. After hot-pressing and spark plasma sintering of cerium-oxide-doped AlN, these two phases have been clearly identified by XRD (see Figure 11 and Figure 12) independently of the CeO_2_ amount. However, CeAl_11_O_18_ only appeared in minute traces. The only sample without this cerium aluminate is composed of 3 wt.% CeO_2_ addition sintered by SPS.

Quenching tests were performed at 1400 °C and 1500 °C (see Figure 13) in order to determine the reaction path leading to the formation of cerium aluminate. XRD analysis performed on quenched samples denotes the formation of cerium aluminate even at 1400 °C. Moreover, Ce_2_O_3_ has been identified as a secondary phase generated during quenching tests. Besides, no remaining traces of alumina have been detected. Quenching was performed on small samples (about 0.5 g), and the observation of minute traces of alumina was not possible with this method.

The formation of cerium sesquioxide denotes a reduction of the introduced CeO_2_ in the system before the reaction of the formation of CeAlO_3_. Then, Ce_2_O_3_ leads to the formation of cerium aluminates such as CeAlO_3_ or CeAl_11_O_18_ [27,28]. Hence, it is possible to propose a reactional path of cerium aluminate formation during AlN sintering following Equations (2)–(4).

(2) $$2{\;\mathrm{CeO}}_{2}\to {\mathrm{Ce}}_{2}O_{3}+\frac{1}{2}O_{2}$$

(3) $${\mathrm{Ce}}_{2}O_{3}+{\mathrm{Al}}_{2}O_{3}\to 2{\;\mathrm{CeAlO}}_{3}$$

(4) $${\mathrm{Ce}}_{2}O_{3}+11{\mathrm{Al}}_{2}O_{3}\to 2{\;\mathrm{CeAl}}_{11}O_{18}$$

**Figure 17:**
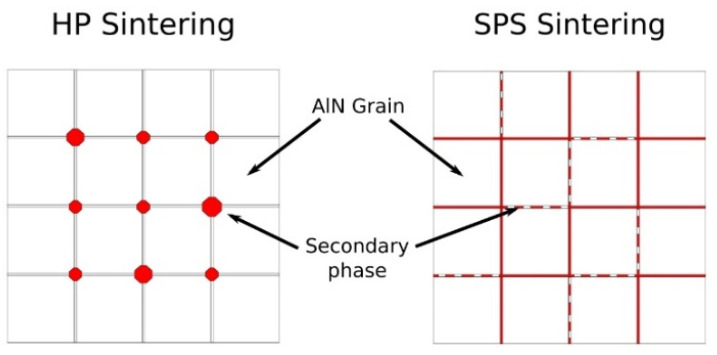
Schematic representation of the microstructure of AlN + CeO_2_ sintered samples obtained by HP and SPS.

Regarding the microstructure of sintered samples in Figure 8 and Figure 9, it appears that SPS sintering seems to improve the global distribution of secondary phases. On HP-sintered samples, secondary phases appeared to be locally distributed on the triple point of the grains. However, on SPS-sintered samples, secondary phases seem to perfectly wet aluminum nitride grains. This global distribution, which appears similar to the expected frozen liquid phase, allowed us to improve the additive particles’ distribution into the global AlN microstructure. Thanks to the SPS process, the secondary phases had insufficient time to coalesce at the triple point and remained around AlN grains. As no amorphous area could be detected by TEM observations (see Figure 10), it confirmed that this liquid-like phase is completely crystallized. The microstructure of sintered samples can be modeled as presented in Figure 17.

During HP or SPS sintering, the reactions of formation did not seem to be influenced by the nature of the densification technic. However, the secondary phases’ distribution is strongly impacted. It evolved from size-grains agglomerated at the AlN triple points to thin cordons forming a quasi-continuous web around AlN grains along grain boundaries (see Figure 9).

In conclusion, during the sintering of aluminum nitride, cerium oxide, introduced as the sintering aid, has been reduced into Ce_2_O_3_ and reacted with the amorphous aluminum hydroxide to form a transient liquid phase. Final secondary phases, such as CeAlO_3_ or CeAl_11_O_18_ in minute traces, are generated and distributed at grain boundaries according to the sintering process. Hot-pressing led to the formation of coalesced phases at the AlN triple points while SPS induced the formation of a thin film around AlN grains. An increase in the CeO_2_ amount enhanced the densification via the generation of a higher volume of secondary phases.

### 4.2. Influence of Secondary Phases on Electrical Property of Ceramics

**Figure 18:**
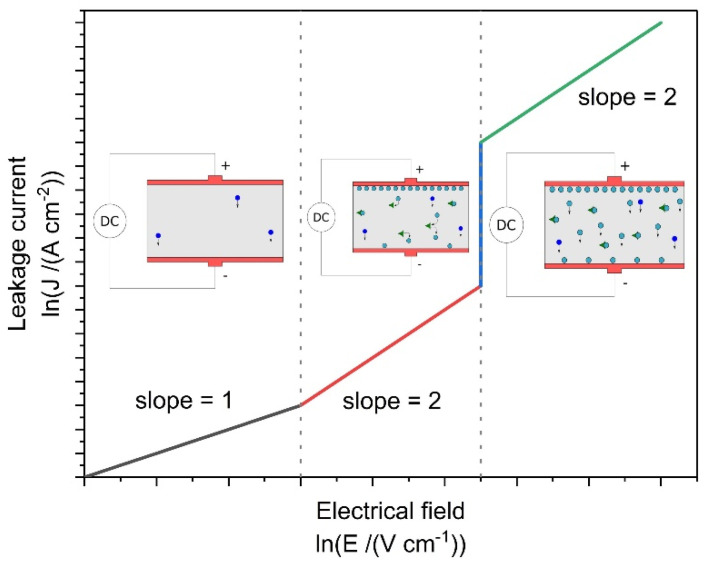
Logarithmic representation of the current–voltage characteristic of a dielectric material according to Space Charge Limited Current mechanism.

The conduction mechanism of AlN ceramics has been determined and corresponds to the Space Charge Limited Current (SCLC) theory [19,20]. This mechanism can be schematized as presented in Figure 18.

In the SCLC model, when a material is placed under a low electrical field (E), the density of current going through (J) is only a function of its free charge carriers. When the field is increased, some electrical charges are injected into the volume of the material. Most of these carriers are stopped by electronic traps within the material and the equilibrium trapping–release participates in the measured current. Finally, under a high electrical field, all traps are statistically filled and injected charges freely increased the measured current. Mathematical equations behind this mechanism justified the use of a log–log representation to discriminate, at best, the three slopes related to this mechanism. The first part, corresponding to an ohmic behavior, follows Equation (5) and must be presented in a log–log graph with a slope equal to 1.

(5) $$J=q\cdot n_{0}\cdot \mu$$

where *q* is the elementary charge, *n*_0_ is the number of intrinsic charge carriers, and *μ* is the charge carrier mobility.

When charges are injected into the ceramic volume, the mechanism can be described according to Equation (6) [19].

(6) $$J=\frac{9}{8}\cdot e_{r}\cdot \epsilon_{0}\cdot \mu \cdot \theta \cdot \frac{E^{2}}{e}$$

where *ε_r_* and *ε_0_* are the material and vacuum dielectric permittivity values, respectively, *θ* is the occupation rate of electronic traps, and *e* is the thickness of the material. When charges have been trapped and released, the *θ* takes a value between (0, 1). When all electronic traps could be considered as fulfilled, *θ* takes a value of 1. Then, the log–log representation leads to slopes equal to 2.

According to Figure 14 and Table 4, it can be assumed that the electrical behavior of sintered samples is attributed to the space charge limited current mechanism thanks to linear representations. The identification of SCLC conduction within aluminum nitride has been presented by Roske et al. [29] and Breit et al. [20]. These authors presented two slopes in the J (E) logarithmic representation. The first slope is close to 1 and the second is between 2.5 and 4.3, and these are attributed to the SCLC mechanism. However, no information was given about the sintering process, microstructure, or eventual secondary phases of samples used.

Regarding the samples sintered by HP, with 1 wt.% of CeO_2_ and 3 wt.% of CeO_2_, respectively, global slopes increased with an increase in temperature. The conductivity is enhanced by the temperature, as is the conduction mode under DC solicitation. An increase in the secondary phase fraction, which for HP sintering can be simulated by an increase in the number and size of secondary grains (see Figure 17), induces a decrease in the global conductivity within the ceramic. Ohmic behavior, which is conserved up to 200 °C for the sample with 1 wt.% of CeO_2_, disappears in favor of a charge injection for the 3 wt.% CeO_2_-doped AlN ceramic even at low temperatures.

For ceramics obtained by SPS, from 50 °C, the conduction behavior has been identified as a charge injection, regardless of the amount of CeO_2_ addition. The two samples obtained by SPS showed a similar electrical response, even if the calculated slopes go up to the theoretical value of 2. However, according to the study of Rose on the space charge limited current in solids [30], it is possible to observe interference due to traps on the current–voltage characteristic. The carriers’ traps concentration will influence the measure and induces a slope value much higher than the theoretical 2 obtained by leakage measurement.

Like HP samples, the electrical conductivity in SPS ceramics is enhanced by temperature. The absolute value of the leakage current increases to reach the measurement limit of the device.

Regarding the influence of the sintering process, it appears that SPS permits one to strongly improve the electrical conductivity of ceramics. The morphology and distribution of the secondary phase induced by SPS modified the conduction mechanism under DC solicitation prior to the influence of the volume fraction of these phases. As a discreet compound included in an AlN microstructure, cerium aluminate CeAlO_3_ grains’ volume decreases the global conductivity of the ceramic.

## 5. Conclusions

During this study, AlN ceramics have been densified by both HP and SPS processes. The presence of an amorphous layer, natively formed around AlN grains, was underlined by extensive characterization of raw powders. During the sintering of aluminum nitride ceramics, it has been shown that this amorphous layer will be transformed into aluminum oxide, such as γ-Al_2_O_3_, δ-Al_2_O_3_, and, finally, α-Al_2_O_3_ up to 1150 °C. The use of CeO_2_ as a sintering aid promoted densification by generating, along with this aluminum oxide phase, a transient liquid phase. The reaction between cerium oxide and aluminum oxide involved the reduced-phase Ce_2_O_3_. The reduction of CeO_2_ into Ce_2_O_3_ appeared to be an intermediate stage in the formation of cerium aluminate. According to XRD observations, correlated by the pseudo-binary Ce_2_O_3_–Al_2_O_3_, secondary phases such as CeAlO_3_ and CeAl_11_O_18_ have been formed during sintering by both HP and SPS. The microstructure is confirmed to be strongly impacted by the sintering process. However, using a selected densification cycle, it is possible to use SPS in order to obtain a secondary phase distribution around AlN grains, and use HP to obtain secondary phases located at the triple points. As underlined by leakage measurements, the conduction path through the sample appeared to be influenced by this phase distribution modification. Hence, the global conductivity of the sample depends not only on the number of secondary phases generated during sintering but also on the spatial distribution of these phases. Optimizing both the additive amount and sintering process, a sample obtained by SPS with 3 wt.% CeO_2_ appeared more conductive by about four orders of magnitude compared to its equivalent obtained by HP.

## Figures and Tables

**Table 1:** Chemical analyses of raw powders.

Elementary Analysis IGA (¤) and GDMS	**AlN Raw Powder**	**CeO_2_ Raw Powder**
	O: 1.10 wt.% (¤)	P: 540 ppm
	H: 0.05 wt.% (¤)	Al: 490 ppm
	Si: 21 ppm	Na: 120 ppm
	Cl: 20 ppm	Fe: 67 ppm
	Fe: 18 ppm	Si: 62 ppm

**Table 2:** Distinctives temperatures of ceramics obtained by both HP and SPS.

Sintering Process	Sample	Beginning of Shrinkage	Maximal Shrinkage
HP	AlN + 1 wt.% CeO_2_	1320 °C ± 30 °C	1660 °C ± 20 °C
	AlN + 3 wt.% CeO_2_	1350 °C ± 20 °C	1680 °C ± 20 °C
SPS	AlN + 1 wt.% CeO_2_	1180 °C ± 20 °C	1590 °C ± 10 °C
	AlN + 3 wt.% CeO_2_	1200 °C ± 20 °C	1520 °C ± 10 °C

**Table 3:** Density of the sintered samples.

Sintering Process	Sample	Density (g cm^−3^)	Relative Density	Open Porosity
HP	AlN + 1 wt.% CeO_2_	3.27	99.8%	< 0.5%
	AlN + 3 wt.% CeO_2_	3.31	99.7%	
SPS	AlN + 1 wt.% CeO_2_	3.27	99.6%	
	AlN + 3 wt.% CeO_2_	3.31	99.5%	

**Table 4:** Slopes calculated from the log–log representation of leakage current measurements obtained at different temperatures.

Sintering Process	Composition	50 °C	100 °C	150 °C	200 °C
HP	AlN + 1 wt.% CeO_2_	1.8	1.9	2.1	2.5
	AlN + 3 wt.% CeO_2_	1.0 (L) 1.6 (H)	1.0	1.2	1.3
SPS	AlN + 1 wt.% CeO_2_	1.9	1.9	2.4 (L) 3.2 (H)	2.5 (L) 3.1 (H)
	AlN + 3 wt.% CeO_2_	2.7	3.0	3.3	-

## Data Availability

Not applicable.
